# The *Citrobacter rodentium* type III secretion system effector EspO affects mucosal damage repair and antimicrobial responses

**DOI:** 10.1371/journal.ppat.1007406

**Published:** 2018-10-26

**Authors:** Cedric N. Berger, Valerie F. Crepin, Theodoros I. Roumeliotis, James C. Wright, Nicolas Serafini, Meirav Pevsner-Fischer, Lu Yu, Eran Elinav, James P. Di Santo, Jyoti S. Choudhary, Gad Frankel

**Affiliations:** 1 MRC Centre for Molecular Bacteriology and Infection, Department of Life Sciences, Imperial College London, London, United Kingdom; 2 Division of Cancer Biology, The Institute of Cancer Research London, London, United Kingdom; 3 Innate Immunity Unit, Institut Pasteur, Paris, France; 4 Inserm U1223, Paris, France; 5 Department of Immunology, the Weizmann Institute of Science, Rehovot, Israel; University of Michigan Medical School, UNITED STATES

## Abstract

Infection with *Citrobacter rodentium* triggers robust tissue damage repair responses, manifested by secretion of IL-22, in the absence of which mice succumbed to the infection. Of the main hallmarks of *C*. *rodentium* infection are colonic crypt hyperplasia (CCH) and dysbiosis. In order to colonize the host and compete with the gut microbiota, *C*. *rodentium* employs a type III secretion system (T3SS) that injects effectors into colonic intestinal epithelial cells (IECs). Once injected, the effectors subvert processes involved in innate immune responses, cellular metabolism and oxygenation of the mucosa. Importantly, the identity of the effector/s triggering the tissue repair response is/are unknown. Here we report that the effector EspO ,an orthologue of OspE found in *Shigella spp*, affects proliferation of IECs 8 and 14 days post *C*. *rodentium* infection as well as secretion of IL-22 from colonic explants. While we observed no differences in the recruitment of group 3 innate lymphoid cells (ILC3s) and T cells, which are the main sources of IL-22 at the early and late stages of *C*. *rodentium* infection respectively, infection with Δ*espO* was characterized by diminished recruitment of sub-mucosal neutrophils, which coincided with lower abundance of Mmp9 and chemokines (e.g. S100a8/9) in IECs. Moreover, mice infected with Δ*espO* triggered significantly lesser nutritional immunity (e.g. calprotectin, Lcn2) and expression of antimicrobial peptides (Reg3β, Reg3γ) compared to mice infected with WT *C*. *rodentium*. This overlapped with a decrease in STAT3 phosphorylation in IECs. Importantly, while the reduced CCH and abundance of antimicrobial proteins during Δ*espO* infection did not affect *C*. *rodentium* colonization or the composition of commensal *Proteobacteria*, they had a subtle consequence on *Firmicutes* subpopulations. EspO is the first bacterial virulence factor that affects neutrophil recruitment and secretion of IL-22, as well as expression of antimicrobial and nutritional immunity proteins in IECs.

## Author summary

*Citrobacter rodentium* is a gold standard model to study pathogen-host-microbiome interactions. Two of the hallmarks of *C*. *rodentium* infection are colonic damage repair responses and colitis; symptoms that are shared with inflammatory bowel diseases in humans. The processes leading to tissue damage repair responses and the implicated bacterial virulence factors are still elusive. In this paper, we show that the *C*. *rodentium* type III secretion system effector EspO plays a major role in triggering damage healing responses, recruitment of neutrophils to the colonic villi, secretion of IL-22 from colonic explants and expression of IL-22 regulated genes in intestinal epithelial cells. This paper is the first to report a bacterial virulence factor that impacts on both intestinal epithelial cell proliferation and immune responses.

## Introduction

*Citrobacter rodentium* is an extracellular, mouse specific, intestinal pathogen used to model mechanisms of virulence employed by the human pathogens enteropathogenic and enterohemorrhagic *Escherichia coli* (EPEC and EHEC) and inflammatory bowel diseases [1]. In C57BL/6 mice, shedding of *C*. *rodentium* peaks around 8 days post infection (DPI) before being cleared, first via IgG opsonization of bacteria expressing virulence factors and phagocytosis by neutrophils and then through competition by the endogenous microbiota [2]. Infection with C. *rodentium* elicits robust tissue repair responses, which are characterized by production of IL-22 and cell proliferation leading to colonic crypt hyperplasia (CCH) [3,4], as well as colitis. Although a number of host pathways involved in CCH have been identified [5,6], the *C*. *rodentium* virulence factor/s implicated in eliciting the tissue repair response remain elusive.

Both innate and adaptive immune responses are vital for *C*. *rodentium* elimination [1]. *C*. *rodentium* and its virulence factors are detected by pathogen recognition receptors (PRRs) such as toll-like receptors (TLR)-2 [7] and TLR-4 [8] and activate both the non-canonical (caspase-11) [9] and canonical (e.g. NLRP3) [10] inflammasome pathways in epithelial and myeloid cells. *C*. *rodentium* infection triggers expression of pro-inflammatory cytokines, e.g. TNF-α, Cxcl-1 (KC), IL-6 and IL-23, which activate innate lymphoid cells (ILCs) and induce differentiation of naïve T helper (Th) cells into Th1, Th17 or Th22 effector cells secreting interferon-γ (IFN-γ), IL-17A and IL-22, respectively [1,11]. IL-22 triggers production of Reg family antimicrobial peptides including Reg3β and Reg3γ in intestinal epithelial cells (IECs) and plays a critical role in maintaining the epithelial barrier and controlling the bacterial burden [12,13]. At an early stage of the infection (4 DPI), ILC3 are the major source of IL-22 [14,15] whereas CD4^+^ T cells secrete IL-22 at a later stage (after 9 DPI) [13]. Importantly, Lee et al. have recently reported that CD11b^+^ Ly6C^+^ Ly6G^+^ neutrophils are also a main source of secreted colonic IL-22 in response to *C*. *rodentium* infection [16].

*C*. *rodentium* colonizes the apical surface of IECs while forming attaching and effacing (A/E) lesions, which are characterized by intimate bacterial interactions with the brush border microvilli [17]. Key to the *C*. *rodentium* infection strategy is the injection of multiple effectors into IECs via a type III secretion system (T3SS). Following translocation, the effectors take control of cell signaling for the benefit of the adherent pathogen, including mitochondrial functions (Map, EspF) [18], apoptosis (NleB, NleH), necroptosis (EspL), cellular trafficking (EspG), phagocytosis (EspJ, EspH, EspF), non-canonical and canonical inflammasome pathways (EspI/NleA, NleF) and innate immune responses (NleC, NleD, NleE, Tir) [19,20]. *C*. *rodentium* also encodes the T3SS effector EspO, an orthologue of OspE found in *Shigella spp* [21]. Importantly, EHEC O157:H7 encodes two EspO paralogs [22]. Recently, we reported that infection of mice with a *C*. *rodentium* mutant lacking the effector EspO (Δ*espO*) results in significantly higher bacterial load from 14 DPI compared to infection with wild type (WT) *C*. *rodentium*, which concur with reduced levels of colonic CD4^+^ T cells and *C*. *rodentium*-specific serum IgG antibodies [23]. In this paper, we report that EspO plays a role in triggering nutritional immunity, cell proliferation, mucosal innate immune responses, phosphorylation of STAT3 and expression of antimicrobial peptides.

## Results

### EspO triggers CCH and cell proliferation

**Fig 1:**
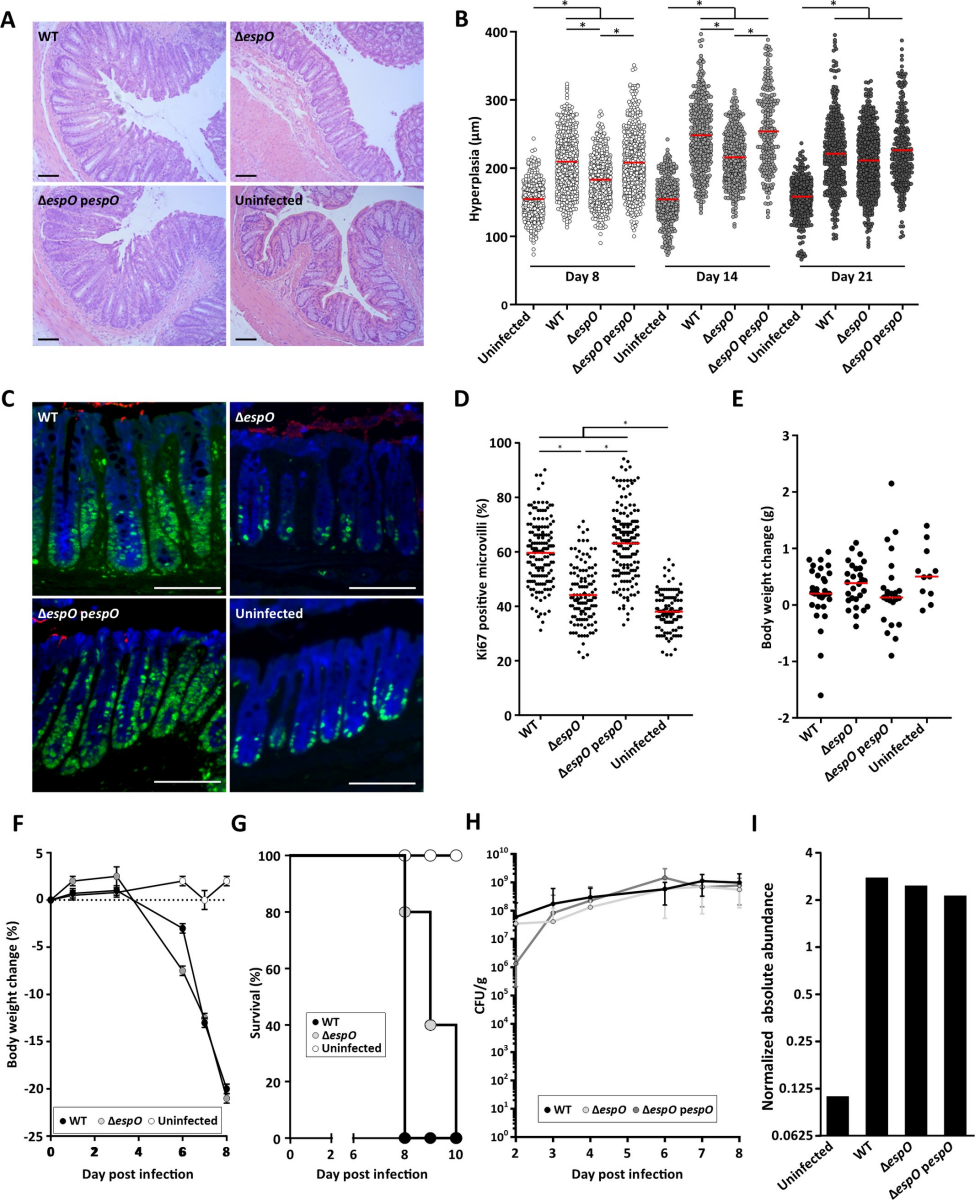
EspO induces CCH and cell proliferation. **(A)** Representative H&E section of colonic tissue 8 DPI with WT, Δ*espO* or *ΔespO*-pespO (n = 17); uninfected mice were used as a control (scale bar 100 μm). **(B)** Measurements of crypt length reveal a significantly reduced level of colonic hyperplasia 8 DPI in mice infected with Δ*espO* compared to WT and *ΔespO*-pespO (*: Kruskal-Wallis test with p-value < 0.05, bars represent mean). **(C)** Representative immunostaining of Ki-67 (green), *C*. *rodentium* (red) and E-cadherin (blue) in colonic section from mice (n = 5) 8 DPI with Δ*espO*, WT or *ΔespO*-pespO; uninfected mice were used as a control (scale bar 100 μm). **(D)** Measurements of Ki-67 staining versus crypt length reveal a significantly decreased level of cell proliferation in mice infected with Δ*espO* compared to WT and the complemented strain (*: Kruskal-Wallis test with p-value < 0.05, bars represent mean). **(E)** Similar body weight was recorded for C57BL/6 mice infected with WT, Δ*espO* or Δ*espO-pespO*; uninfected mice were used as a control (n > 12). **(F)** Similar body weight loss was recorded in Rag2^-/-^ il2rg^-/-^ mice infected with WT or Δ*espO*; uninfected mice were used as a control (n = 5). **(G)** Rag2^-/-^ il2rg^-/-^ mice survival over time. Mice infected with Δ*espO* showed a small increase of survival compare to mice infected with WT *C*. *rodentium* (n = 5). **(H)** Fecal *C*. *rodentium* CFUs overtime. WT *C*. *rodentium*, Δ*espO* and Δ*espO-pespO* similarly colonized C57BL/6 mice up to 8 DPI. **(I)** Normalized abundance of IECs-associated *C*. *rodentium* proteins following infection with WT, ΔespO and the complemented strains.

Infection of C57BL/6 mice revealed that at 8 and 14 DPI, *C*. *rodentium* Δ*espO* triggered reduced CCH (48% and 40% reduction, respectively) compare to WT; this phenotype was fully complemented with a plasmid encoding EspO (p*espO*) (Fig 1A and 1B). By 21 DPI, no difference in the level of CCH was recorded between mice infected with WT, Δ*espO*-p*espO* or Δ*espO* (Fig 1B).

Ki-67 is a marker of cell proliferation expressed in all phases of active cell cycle but absent in resting cells [24]. Consistently, significant reduction (72% reduction) in Ki-67 staining was seen in mice infected with Δ*espO* compared to mice infected with WT or the complemented strain at 8 DPI (Fig 1C and 1D). Importantly, no difference in body weight was observed between mice infected with WT, Δ*espO* and Δ*espO*-p*espO* at 8 DPI and the uninfected control mice (Fig 1E).

As Δ*espO* cause a significantly reduced cell proliferation, a marker of tissue damage repair, we determined the outcome of infection of the highly susceptible Rag2^-/-^ il2rg^-/-^ mice (n = 5), which lack NK, ILCs, T and B cells. This revealed that while both WT and Δ*espO* triggered a similar decline in body weight (Fig 1F), there was a small delay in mortality in mice infected with the Δ*espO* (Fig 1G), which is consistent with the mutant causing less colonic damage.

### The effect of EspO on cellular metabolic processes

A previous study has recently shown that *C*. *rodentium* utilizes the T3SS to induce CCH as a means to oxygenate the mucosa and to drive bacterial expansion via CydAB-mediated aerobic respiration [25]. Enumeration of colony-forming units (CFUs) per gram of feces revealed that despite the significant difference in CCH there was no difference in bacterial shedding between WT, Δ*espO* and Δ*espO*-p*espO* up to 8 DPI (Fig 1H). As *C*. *rodentium* T3SS effectors execute their function by altering cell signaling in IECs, we aimed to investigate the intracellular processes affected by EspO. To this end, we compared the proteomes of IECs purified from mice infected with WT, Δ*espO* and Δ*espO*-p*espO* at 8 DPI. Assessing purity by flow cytometry revealed that the IEC preparations were enriched by over 90% [26]. For the proteomic analysis, we only considered changes to protein abundance between WT and Δ*espO* if they were fully restored upon Δ*espO* complementation.

Associated with the IEC proteomes were 773 *C*. *rodentium* proteins (S1 Table), including structural (e.g. EscN, EspA,B,D), chaperone (CesF, CesT) and T3SS effector (Tir, EspF, EspH, EspM2, EspI/NleA) proteins, intimin, the global regulator of virulence RegA [27] and the essential periplasmic serine protease HtrA [28]. Consistent with the similar bacterial shedding (Fig 1C), the proteomes of infected IECs revealed similar abundances of *C*. *rodentium* proteins across the challenged groups (Fig 1I).

**Fig 2:**
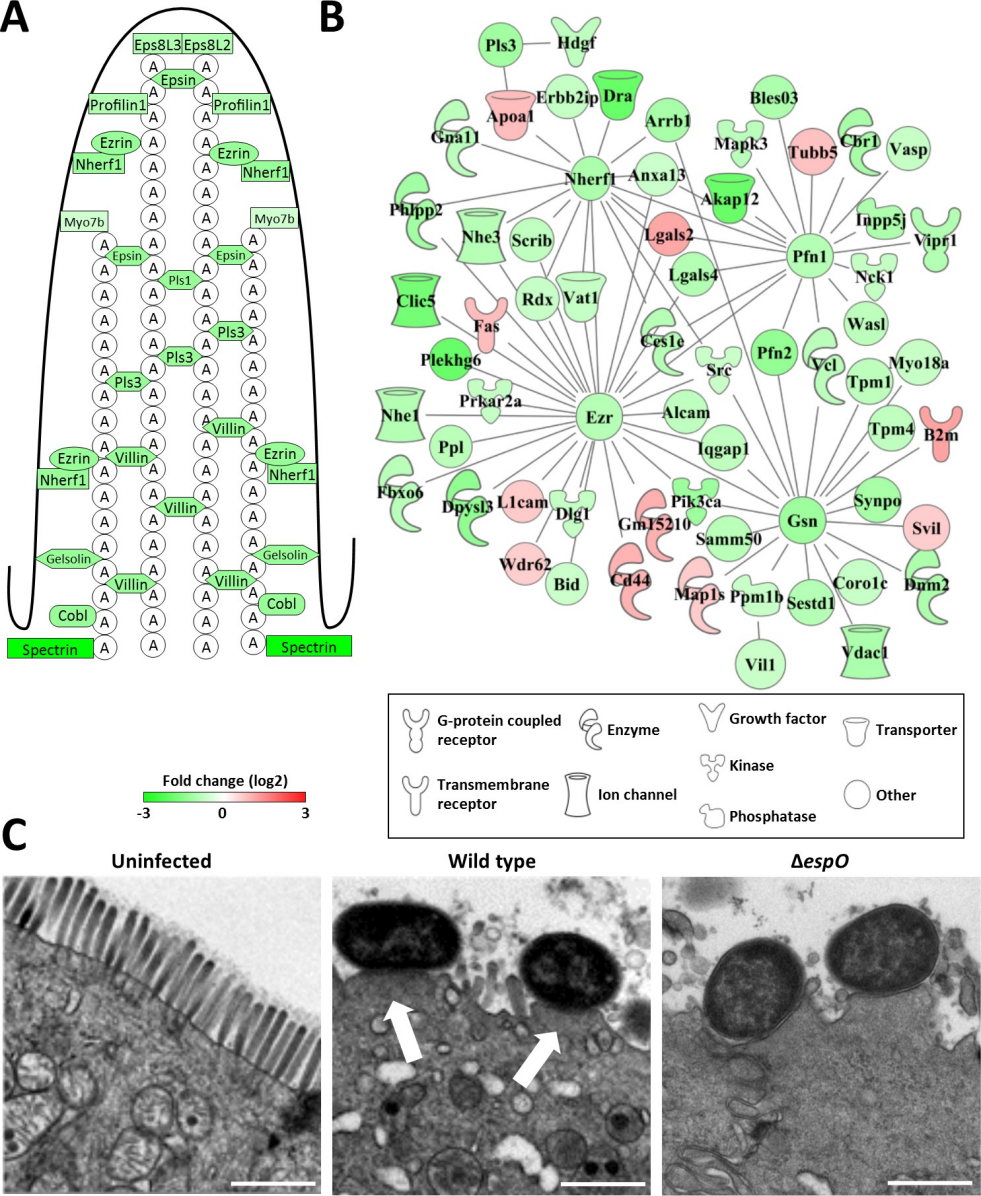
The A/E lesion signature of *C*. *rodentium* infection. **(A)** Schematic representation of a microvillus with structural protein abundance 8 DPI. **(B)** Interaction network of brush border related proteins 8 DPI. **(C)** TEM micrographs of uninfected and infected IECs, showing A/E lesions on 8 DPI (arrows, scale bar 1 μm).

As key to the *C*. *rodentium* infection strategy is formation of A/E lesions, which has a major impact on the shape and function of IECs, we first compared the abundance of brush border proteins in infected IECs. This revealed that Eps8, Villin, Plastin, Ezrin and Espin, as well as actin binding proteins Profilin, Gelsolin, Cobl and Spectrin (and their associated proteins) were in similarly lower abundance in the infected groups compared to the uninfected control mice (Figs 2A, 2B, S1A and S1B). These findings were confirmed by observations made in transmission electron microscopy (TEM) showing typical A/E lesions in colons infected with either the WT or Δ*espO* (Fig 2C).

**Fig 3:**
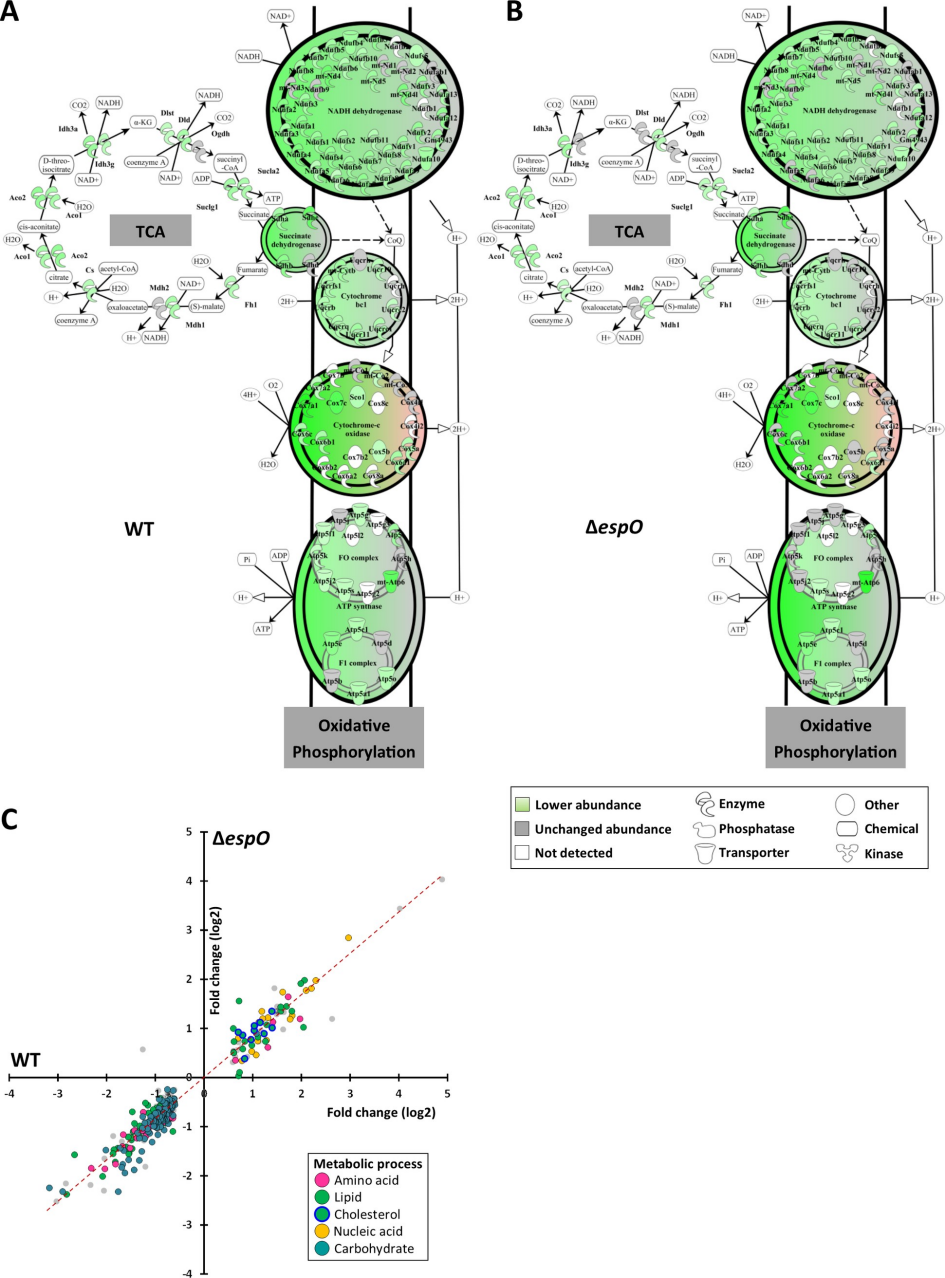
Metabolic changes are independent of CCH. **(A)** and **(B)** Schematic representation of the mitochondrial TCA cycle and respiratory chain showing a similar protein abundance in IECs infected with WT and Δ*espO*. **(C)** Scatter plot of the abundance (log2 fold change) of the metabolic enzyme in IEC infected with WT (x axis) and Δ*espO* (y axis).

We have recently reported that 1,447 proteins, mostly associated with metabolic processes (e.g. ATP production in the mitochondria, lipid biogenesis), were in lower abundance in *C*. *rodentium*-infected IECs. In contrast, *C*. *rodentium* infection induced the creatine and cholesterol biogenesis pathways, as well as cholesterol efflux [18]. As cell proliferation is dependent on metabolic activity and ATP consumption, we compared the abundance of the metabolic enzymes between WT- and Δ*espO-*infected IECs. This revealed a similar profile in the infected IECs, with decreased abundance of proteins in the TCA cycle, oxidative phosphorylation (Fig 3A and 3B) and lipid metabolism (Fig 3C), and increased abundance of proteins in the cholesterol, creatine and nucleic acid metabolism (Fig 3C).

Taken together, these results suggest that cellular processes involved in effacement of the brush border microvilli, disruption of the mitochondria and cell metabolisms are not related to CCH. Moreover, the data show that CCH is dispensable for proliferation of *C*. *rodentium in vivo*.

### EspO triggers expression of antimicrobial peptides

**Fig 4:**
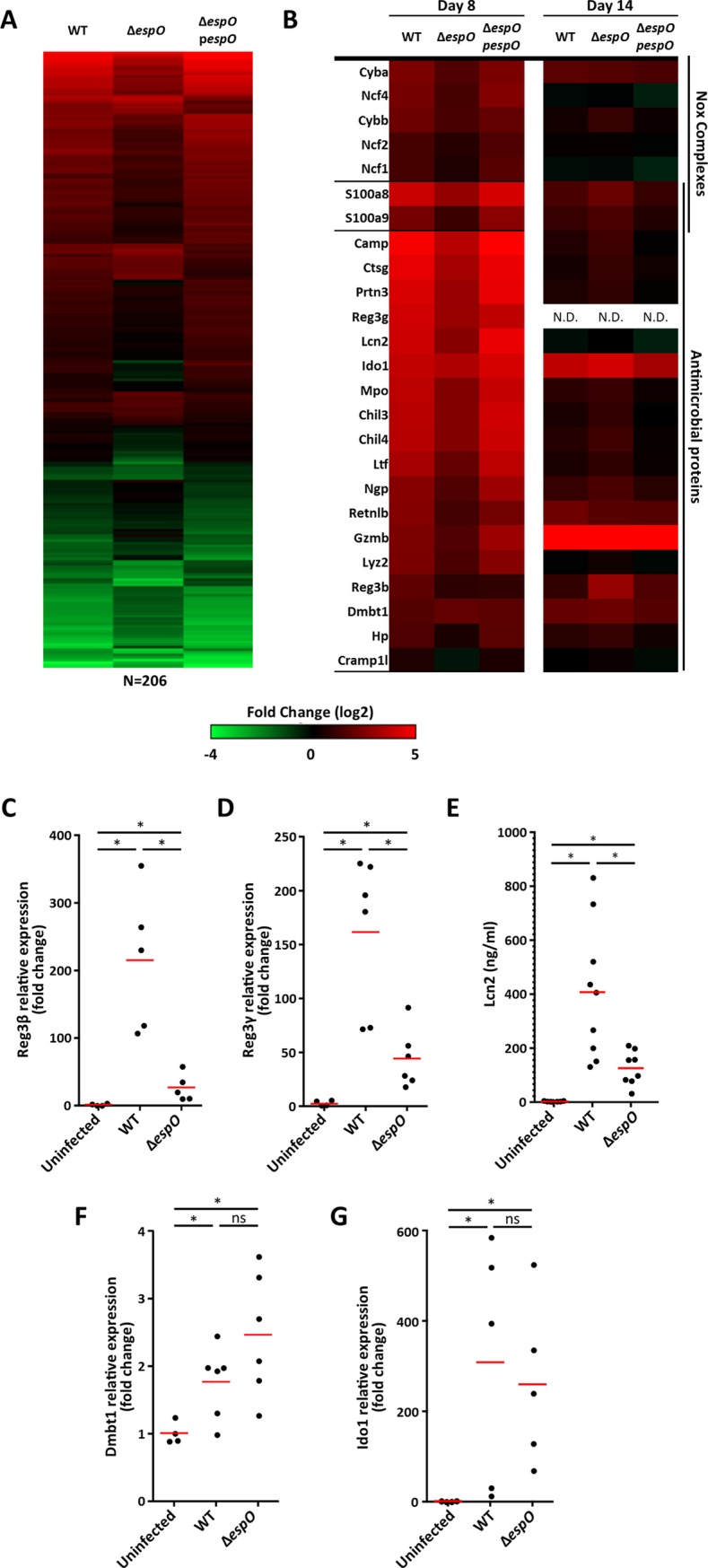
EspO alters expression of AMPs. **(A)** Heatmap of the 206 EspO-specific changing proteins. Abundance changes in IECs proteomes are comparing infected to uninfected mice. **(B)** Heatmap of differentially expressed Nox and AMPs 8 and 14 DPI. **(C)** and **(D)** Fold change in *reg3β* and *reg3γ* mRNA expression levels. **(E)** Fecal Lcn-2 measured by ELISA. **(F)** and **(G)** Fold change in *dmbt1* and *ido1* mRNA expression levels (*: Kruskal-Wallis with p-value < 0.05, each dot represents an individual mouse and bars geometric mean).

In order to determine which biological processes are affected by EspO, we searched for proteins with differential abundance in WT- and Δ*espO-*infected IECs. This revealed 206 EspO specific proteins with altered abundance compared to WT and Δ*espO*-p*espO* (Fig 4A; S2 Table). Among the EspO specific proteins, 41 were grouped under the GO term defense and reactive oxygen species (ROS) responses. Expression of the Nox2 system (e.g. Ncf1, Ncf2, Ncf4, Cyba) was induced in IECs infected with WT, but to a significantly lesser extent in IECs infected with Δ*espO* (Fig 4B). Nox2 activity is under the regulation of the calprotectin (a heterodimeric complex made of S100a8 and S100a9), which is translocated to the plasma membrane upon activation [29]. Whereas both S100a8 and S100a9 were in higher abundance in IECs infected with WT and Δ*espO*-p*espO*, they were in lower abundance in the Δ*espO-*infected IECs (Fig 4B). As ROS and calprotectin have an antimicrobial activity, we extended the comparison to other antimicrobial proteins (AMPs) expressed in IECs infected with WT, Δ*espO* and Δ*espO*-p*espO* 8 DPI and 14 DPI. This revealed elevated abundance of 16 AMPs, including the antimicrobial peptides Reg3β and Reg3γ, lysozyme (Lyz2) and proteins involved in nutritional immunity including lactotransferin (Ltf, involved in binding and transport of iron), Lipocalin-2 (Lcn2, targeting the bacterial ferric-siderophore enterobactin), as well as calprotectin (which sequesters Mn and Zn), in WT- and Δ*espO*-p*espO-*infected mice 8 DPI; compared to WT, these AMPs were found in significantly lower abundance following infection with Δ*espO* (Fig 4A). The abundance of these proteins, with the exception of Camp, Ctsg and Reg3β, decreased at 14 DPI, with no significant difference between WT and Δ*espO*. Importantly, several of the identified AMPs were thought to be expressed only by immune cells (Lyz2, S100a8, S100a9, Mpo, Ctsg). However, recent studies have shown that these AMPs are expressed by other cells types upon cytokines stimulation: Lyz2 is expressed by crypt and Paneth cells [30], while S100a8 and S1009 are produced by epithelial cells [31]. In addition, others AMPs, associated with neutrophil extracellular traps (NET), could be found on the surface of IECs (e.g. Mpo, Ctsg) [32].

In order to validate the proteomics data, we quantified the levels of the Reg3β and Reg3ϒ mRNA in IECs by qPCR and fecal Lcn2 by ELISA. We used Dmbt1 (a glycoprotein of the scavenger receptor cysteine-rich family targeting bacteria and reducing their adhesion to the cells) and Indoleamine 2,3-dioxygenase 1 (Ido1, mediates tryptophan depletion and increases antimicrobial metabolites including kynurenine and 3-hydroxy-kynurenine [33]) as controls. Whereas the mRNA levels of Reg3β and Reg3ϒ increased significantly following infection with both WT and Δ*espO* compared to control mice, the increase seen in the Δ*espO* was significantly lower compared to WT (Fig 4C and 4D). A similar trend was detected for the abundance of fecal Lcn2, quantified by ELISA (Fig 4E). In contrast, the level of Dmbt1 and Ido1 mRNA increased significantly after infection, with no difference between WT and Δ*espO* (Fig 4F and 4G). Taken together, these data show that EspO affects expression of antimicrobial peptides and nutritional immunity proteins in IECs and suggest the changes observed at the proteome may be partially regulated at the transcriptional level.

### EspO modulates submucosal neutrophils recruitment and secretion of IL-22

**Fig 5:**
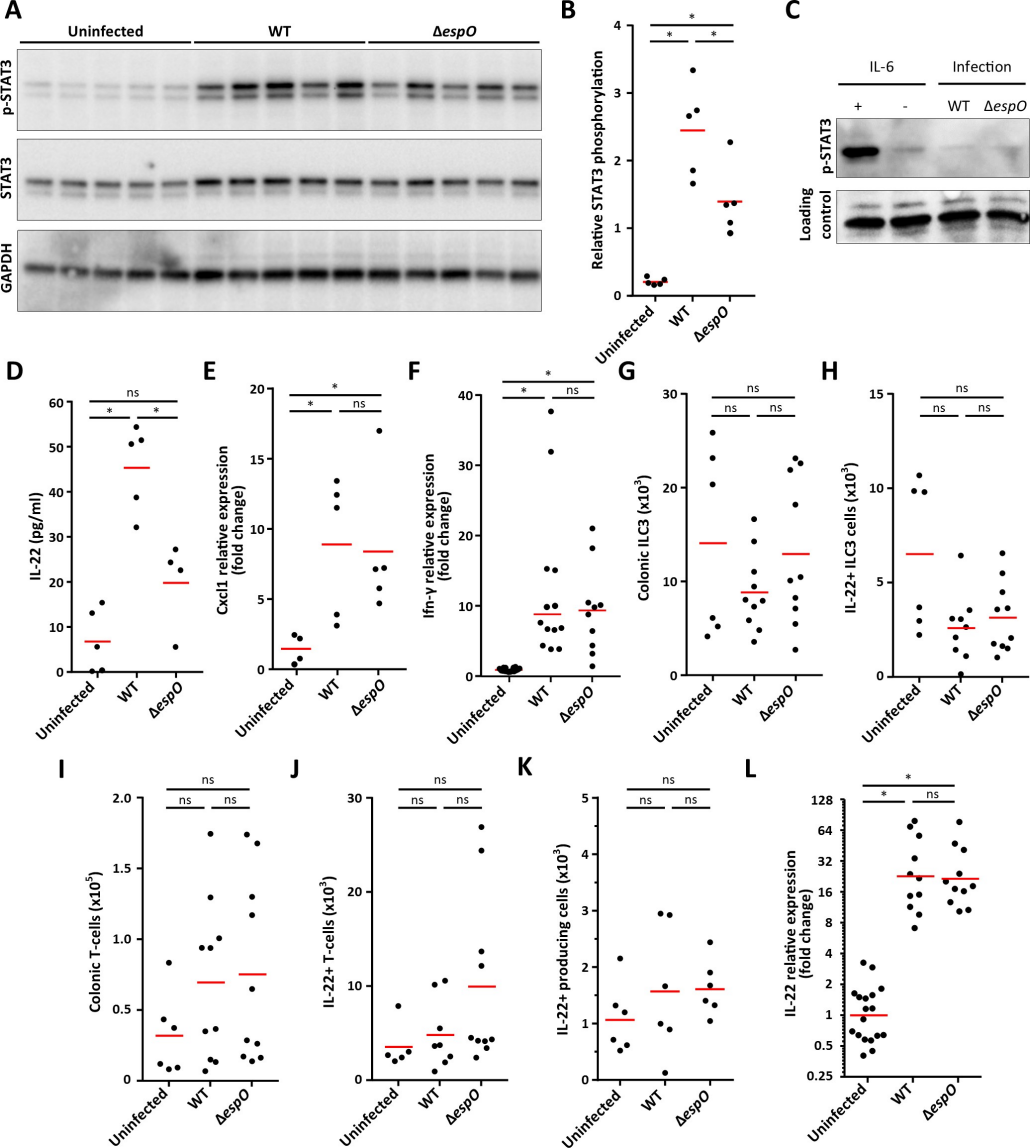
EspO triggers IL-22 release from colonic explants and STAT3 phosphorylation in IECs. **(A)** Western blot showing STAT3 phosphorylation, STAT3 and GAPDH levels in IECs from uninfected, WT- and Δ*espO*-infected mice (5 mice/group). **(B)** pSTAT3 quantification (densitometry) from individual mice. (*: Kruskal-Wallis with p-value < 0.05, each dot represents and individual mouse and bars mean). **(C)** Western blot showing STAT3 phosphorylation levels in uninfected, WT- and Δ*espO*-infected HeLa cells. IL-6 was used as a positive control. No STAT3 phosphorylation was observed during infection. **(D)** IL-22 secretion measured by ELISA from colonic explants from uninfected, WT- and Δ*espO*-infected mice (*: Kruskal-Wallis with p-value < 0.05, each dot represents and individual mouse and bars mean). **(E)** Fold change in *cxcl-1* mRNA expression level in IECs from uninfected, WT- and Δ*espO*-infected mice (*: Kruskal-Wallis with p-value < 0.05, each dot represents an individual mouse and bars geometric mean). **(F)** Fold change in *ifn-γ* mRNA expression level in tissue from uninfected, WT- and Δ*espO*-infected mice (*: Kruskal-Wallis with p-value < 0.05, each dot represents an individual mouse and bars geometric mean). **(G)** and **(I)** Bar graphs indicate the absolute numbers of colonic ILC3 (CD45.2+ CD3—CD5—CD127+ RORγt+ KLRG1—cells) and T cells (CD45.2+ CD3+ CD5+ cells). **(H)** and **(J)** Quantification of IL-22 producing ILC3 and T-cells isolated from infected colons after cytokines and PMA/Ionomycin restimulation. **(K)** Quantification of other IL-22 producing cells isolated from infected colons after cytokines and PMA/Ionomycin restimulation. **(L)** Fold change in *il-22* mRNA expression level in IECs from uninfected, WT- and Δ*espO*-infected mice (*: Kruskal-Wallis with p-value < 0.05, each dot represents an individual mouse and bars geometric mean).

The transcription factor STAT3 commonly regulates expression of genes encoding AMPs, proteins involved in ROS production [34] and cell proliferation [35]. To determine if STAT3 may be differentially activated in IECs infected with WT compared to Δ*espO*, STAT3 phosphorylation on Tyr 705 was accessed by western blotting. Total STAT3 and GAPDH were used as loading controls. Whereas, little STAT3 phosphorylation was observed in uninfected IECs, robust phosphorylation was detected in IECs infected with WT. Importantly, lower level of STAT3 phosphorylation was observed in IECs infected with Δ*espO* (Fig 5A and 5B). No difference in the levels of total GAPDH was detected in the different mice whereas a small increased of total STAT3 is detected during infection. In a control experiment we determined if EspO itself can induce phosphorylation of STAT3. For this, HeLa cells were infected with WT or Δ*espO* for 3 h and the level of STAT3 phosphorylation was measured by WB. IL-6 was used as a positive control. While IL-6 induced strong STAT3 phosphorylation, no phosphorylation was observed in cell infected with either the WT or Δ*espO* (Fig 5C), suggesting that STAT3 phosphorylation is not a direct cell response to infection.

Secreted by immune cells, IL-22, which is a key cytokine needed to control *C*. *rodentium* infection [12], triggers STAT3 phosphorylation and expression of AMPs [36]. IL-22 also plays a role in cell proliferation [37]. We therefore tested if IL-22 secretion into supernatants of colonic explants was reduced in explants that were previously infected with either *C*. *rodentium* wild-type or Δ*espO*. This revealed that levels of IL-22 were 57% lower in mice infected with Δ*espO* (Fig 5D). In order to determine if the EspO influences expression of other cytokines, expression of Cxcl-1 produced by IECs and Ifn-γ produced by immune cells (e.g. natural killers, macrophages and T cells) was analyzed by qPCR in IECs and whole colonic tissue respectively. Whereas Cxcl-1 (Fig 5E) and Ifn-γ (Fig 5F) were induced during *C*. *rodentium* infection, no difference was observed between WT and Δ*espO* 8 DPI, suggesting that EspO selectively alters IL-22 related inflammation.

ILC3, at the early stage of the infection, and Th22 cells, at the later stage of the infection, are the major sources of IL-22. Moreover, neutrophils have also been shown to be an important source of IL-22 during *C*. *rodentium* infection [16]. To identify the immune cell types responsible for IL-22 secretion, colonic immune cells populations were analyzed 8 DPI by FACS. Unexpectedly, no difference was observed in the total number of colonic ILC3 and T cells (Figs 5G, 5I and S2), or in the number of IL-22 producing ILC3 and T cells (Fig 5H and 5J). Furthermore, no difference in other cell types producing IL-22 upon *ex-vivo* stimulation was seen between uninfected and infected colons (Fig 5K). This suggests that EspO did not influence the abundance of IL-22-producing cells, but instead affected the expression or release of IL-22 or the positioning of IL-22-producing immune cell with respect to the colonic mucosal surface. To test this hypothesis, IL-22 mRNA level was analyzed by qPCR and whole colonic tissue. Whereas IL-22 expression was strongly induced during *C*. *rodentium* infection, no difference was observed between WT and Δ*espO* 8 DPI (Fig 5L), suggesting that EspO alters either the release of IL-22 by immune cells or theirs localization in the tissue.

**Fig 6:**
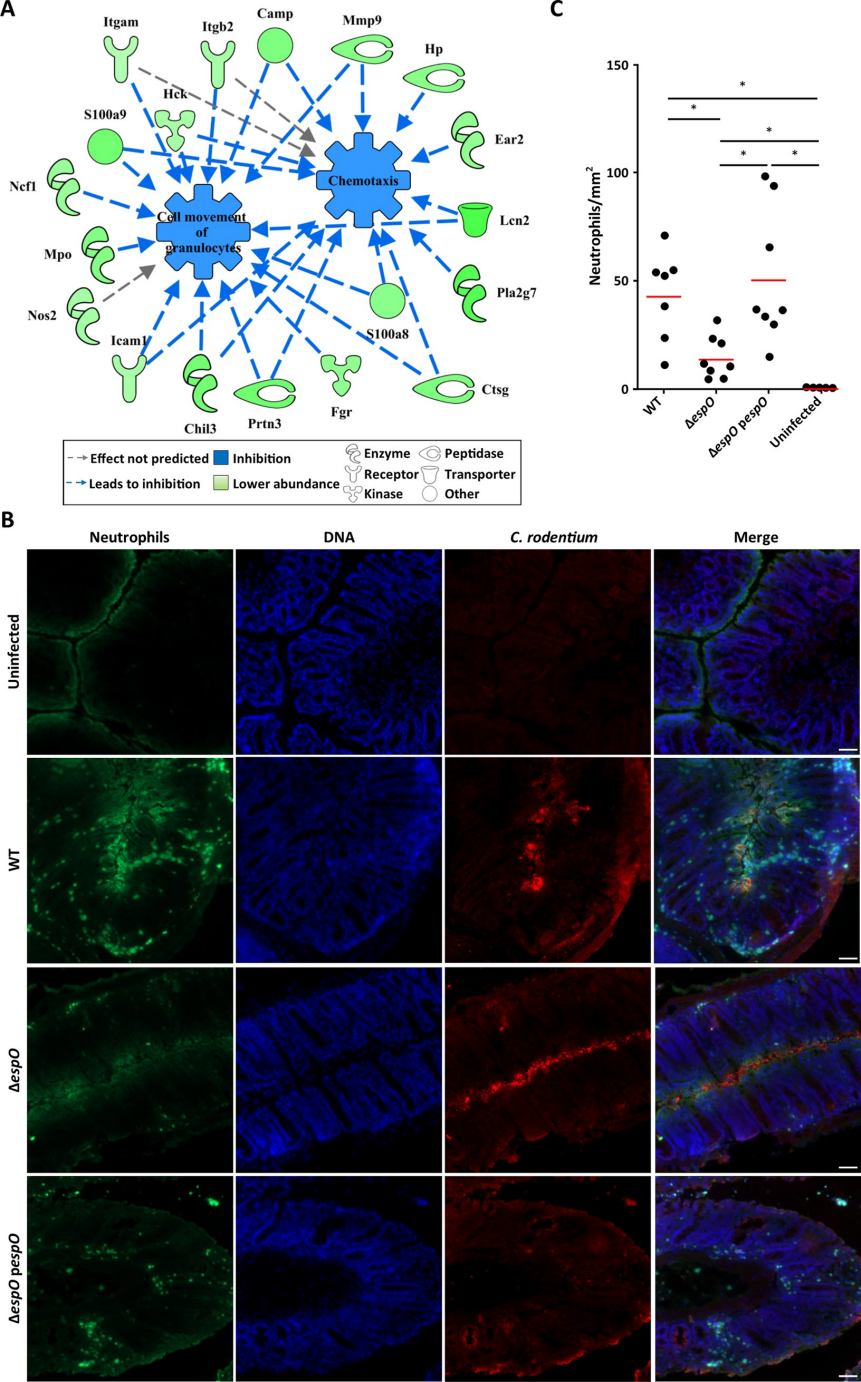
EspO promotes neutrophile transmigration. **(A)** Network analysis illustrating the impact of Δ*espO*-regulated IECs’ proteins on chemotaxis, cell movement of granulocytes. **(B)** Representative immunostaining of neutrophils (Ly6G, green), *C*. *rodentium* (red) and DNA (blue) on colonic section from mice infected with WT, Δ*espO* and Δe*spO*-p*espO* and uninfected mice at day 8 DPI (n = 8) (scale bar 100 μm). **(C)** Quantification reveals a significantly decreased number of neutrophils recruited to the site of infection in mice infected with Δ*espO* compared to WT and the complemented strain (*: Kruskal-Wallis test with p-value < 0.05, bars represent mean).

The proteomics analysis predicted that compared with WT infection, Δ*espO* induces reduced chemotaxis (activation score: -2.987; p-value 3.26 x 10^−4^), cell movement of granulocytes (activation score: -2.8; p-value 2.04 x 10^−7^) and immune response of neutrophils (activation score: -2.543; p-value 3.30 x 10^−5^) (Fig 6A). This is consistent with the predicted lower ROS production as well as the lower abundance of S100a8 and S100a9, which promote chemotaxis [38]. In addition, Mmp9, which digests extracellular matrix and opens tight junction allowing neutrophils transmigration [39] as well as Icam1, a neutrophil ligand which promotes their adhesion to the epithelial cells [40], were in higher abundance in IECs infected with WT and the Δ*espO-pespO* but to a lesser extent in Δ*espO* (Fig 6A).

Recently, neutrophils have been shown to be a main source of secreted IL-22 in the colon during *C*. *rodentium* infection [16] and colitis [31]. While we previously reported no global difference in total neutrophil numbers within inflamed tissue during infection with WT or Δ*espO* [23], supporting our IL-22 expression data (Fig 5K), neutrophil distribution within the inflamed colon was not assessed and could be altered after infection with Δ*espO*. To test this, colonic sections from mice infected with WT, Δ*espO* and Δ*espO-*p*espO* were stained with antibodies against Ly6G, *C*. *rodentium* and Hoechst stain (nuclei). Uninfected sections were used as control. While the number of granulocytes infiltrated into the tissue increased during infection (Fig 6B and 6C), the number was significantly lower in mice infected with Δ*espO* (68% reduction), suggesting that EspO affected neutrophils transmigration toward the site of bacterial attachment. These results suggest that by signaling inside IECs, EspO impacts on neutrophils chemotaxis to the site of *C*. *rodentium* colonization.

### Deletion of *espO* affects *C*. *rodentium* induced dysbiosis

**Fig 7:**
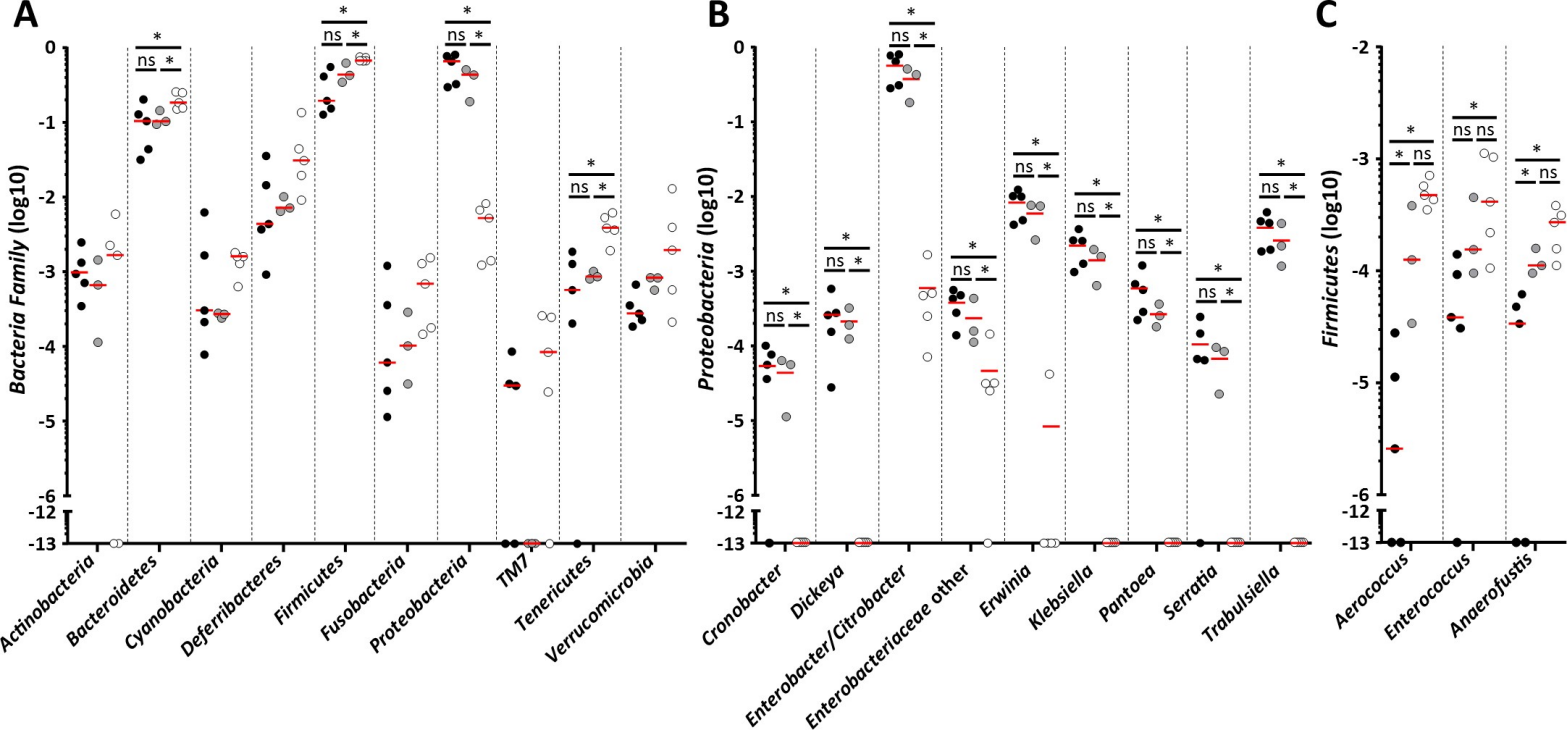
Expression of EspO affects the composition of *Firmicutes* subpopulation. **(A)** Changes in abundance of tissue-associated Phylum between uninfected, WT- and Δ*espO*-infected mice. **(B)** Changes in abundance of tissue-associated *Proteobacteria* between uninfected, WT- and Δ*espO*-infected mice. **(C)** Changes in abundance of tissue-associated *Firmicutes* between uninfected, WT- and Δ*espO*-infected mice. Each dot represents an individual mouse and bars mean (*: Kruskal-Wallis & Mann-Whitney test with FDR corrected p-value < 0.05).

As CCH, neutrophil recruitment and AMPs affect the composition of the gut microbiota, we hypothesized the deletion of *espO*, which affects these parameters, will impact on the nature of dysbiosis induced by *C*. *rodentium*. To test this, we compared the composition of tissue-associated microbiota between mice infected with WT and Δ*espO* 8DPI using 16S RNA sequencing. Whereas *C*. *rodentium* infection induced a dysbiosis with a decreased of *Bacteroidetes*, *Firmicutes* and *Tenericutes* and a proliferation of *Proteobacteria*, no difference was observed at the Phylum level between mice infected with WT or Δ*espO* (Fig 7A). As expansion of *C*. *rodentium* in the colon has been linked to CCH [25], we tested if infection with Δ*espO* affects at the abundance of other *Enterobacteriaceae* (Fig 7B). Consistent with the similar level of shedding, the level of *Citrobacter/Enterobacter* were similar in mice infected with either WT or Δ*espO* (Fig 7B). Moreover, the abundance of other genera was similar in the infected mice, suggesting that proliferation of *Enterobacteriaceae* is independent of CCH and is not affected by the abundance of AMPs. As Reg3γ specifically kills Gram-positive bacteria [41], we analyzed the composition of tissue associated *Firmicutes*. Whereas, the abundance of most of the genera was similar in mice infected with either WT or Δ*espO*, the abundance of *Aerococcus*, *Enterococcus* and *Anaerofuctis* differ between the two infections, with the level in the Δ*espO* infected mice similar to that seen in the uninfected control mice (Fig 7C). This is consistent with a previous report showing that *Reg3*-/- mice have similar numbers of mucosa-associated bacteria belonging to the Gram-negative *Bacteroidetes* phylum and an increase of some *Firmicutes* (*Eubacterium*) [42].

Taken together, these results suggest that by signaling inside IECs, EspO impacts on chemotaxis of neutrophils to the site of *C*. *rodentium* adhesion, secretion of IL-22, phosphorylation of STAT3, cell proliferation and expression of AMPs, which leads to a specific alteration in the abundance of particular *Firmicutes* genera.

## Discussion

One of the main hallmarks of infection with *C*. *rodentium* is induction of tissue damage repair responses, i.e. elaboration of IL-22, secretion of AMPs and proliferation of transit amplifying cells [43]. In this study, we identified for the first time a T3SS effector, EspO, which once injected into infected IECs induces both processes. This raises the following question: what benefit *C*. *rodentium* gains from triggering tissue damage?

EspO is one of the smallest *C*. *rodentium* effectors (10 kDa), yet its deletion has profound effects on how the gut responds to infection. Induction of CCH is a complex event, involving a large number of biological pathways that are triggered directly by *C*. *rodentium* and by the host in response to the infection. Wnt signaling has been previously implicated in *C*. *rodentium*-induced CCH [44]. Roy et al. have shown that in Swiss–Webster mice, *C*. *rodentium* infection induces accumulation of β-catenin during the cell proliferation phase [5]. Our proteomics analysis did not reveal any hallmarks of the Wnt signaling pathway in infected C57BL/6 mice; the abundance of β-catenin was similar to the uninfected mice and none of the known Wnt target proteins were affected. Therefore, our data suggest that EspO delivered into IECs at the site of infection affects cell proliferation in a Wnt-independent fashion.

A triple *C*. *rodentium* mutant (Δ*espH* Δ*cesF* Δ*map*) has been recently reported to colonize the colonic mucosa at a significantly lower level than WT, which was mirrored by reduced CCH and Ki-67 staining. It was proposed that *C*. *rodentium* induces CCH as a means for oxygenation of the mucosal surface, which sustains the preference of the pathogen for aerobic metabolism and promotes pathogenesis [25]. However, the cause and effect relationship between the reduced colonization and lower CCH remained unresolved. Here, we show that that deletion of *espO* also caused significant reduction in CCH, yet colonization of the mucosal surface was unaffected, suggesting an independence of these two processes. Indeed, we have recently shown that *C*. *rodentium* infection diverts ATP production from the mitochondria to glycolysis, which was associated with production of phosphocreatine. In this study, we found that while colonizing at the same levels, WT and Δ*espO* trigger similar changes to metabolism in IECs, including disruption of the mitochondria, suggesting that *C*. *rodentium* is able to extract oxygen independently of CCH.

Induction of CCH seems to be influenced by inflammatory responses to infection. Indeed, deletion of aquaporin-3, an IECs’ basolateral water channel which mediates uptake of H_2_O_2_, has been shown to attenuate ROS responses, reduce CCH and impair *C*. *rodentium* clearance [45], resembling the Δ*espO* phonotype. The abundance of proteins linked to the production of ROS was lower in IECs infected with Δ*espO*. This suggests the existence of a strong link between CCH, ROS and bacterial survival. Moreover, mice infected with *C*. *rodentium* have an increased level of serotonin [46]. Interestingly, rectal injection of serotonin (5-hydroxytryptamine) in rat induced expression of Nox2, production of ROS, neutrophils recruitment and an increase of colonic wall thickness similar with *C*. *rodentium* infection in mice [47]. The exact relation between ROS production, CCH and neutrophils recruitment is still unclear, but it is likely linked to the secretion of chemokines [48].

To date, little is known about the interaction between neutrophils and *C*. *rodentium*. One reason for this is that these cells are short lived and mostly transcriptionally inactive upon their arrival to the site of infection. While the purity of our IECs was greater than 90%, our proteomes contained proteins reported to be neutrophils specific. This is mainly due to incorrect protein annotation, as most studies have examined protein contents in resting cells and not in the context of an inflamed tissue; nonetheless, we cannot exclude the possibility that during the purification process we co-purified proteins from neutrophil NETs. While NETs have been shown to be induced by *E*. *coli*, it is yet unknown if they play a role in controlling *C*. *rodentium* infection.

Neutrophils migration from the microcirculation to the tissues is a multistep process, which remains largely uncharacterized. It involves the Tlr4—Myd88 axis and requires chemokines (e.g. Ccl-3, Cxcl-1), matrix metalloproteinase (MMP) activation and lipids (lipid leukotriene B4). While the overall number of colonic neutrophils was similar between mice infected with WT or Δ*espO*, their tissue penetration was altered. We found similar level of Cxcl-1 expression in IECs, suggesting that EspO does not affect this step. Mice deficient in IECs’ Myd88 are unable to control *C*. *rodentium* infection, as they cannot induce epithelial repair response that maintains the protective barrier, production of neutrophil chemokines and an efficient adaptive immune response. While these mice succumb to *C*. *rodentium* infection, they do not show signs of CCH. Importantly, bone marrow transplantation from WT mice into Myd88-/- mice restored CCH [6]. In addition, the T3SS effector Tir, in a process dependent on Y451 and Y471, has been shown to modulate secretion of Cxcl-1 and recruitment of neutrophils 14 DPI [26]. Importantly, while exhibiting a similar level of shedding, mice infected with *C*. *rodentium* expressing Tir Y451A/Y471A presented reduced CCH 14 DPI. More recently, Cxcl-5 has been shown to mediate neutrophil infiltration during cancer or infection however, it requires further processing post secretion from epithelial cells. Mmp2 and Mmp9 have been shown to cleave Cxcl-5 and enhances it chemotactic activity [49]. Whereas we did not detect a significant increase of the abundance of Mmp2, the abundance Mmp9 increase 4-fold in IECs infected with WT, but only 2 fold in IECs infected with Δ*espO*. It is possible that by modulating the secretion of Mmp9, EspO indirectly modulates Cxcl-5 processing and neutrophil infiltration. Taken together, these results reinforce the link between neutrophils and CCH. However, the mechanism by which neutrophils contribute towards CCH remains unknown. Recent studies have shown that IL-22 can be secreted by neutrophils [16,31], however infection of IL-22 deficient mice with *C*. *rodentium* results in greater CCH [12].

Expression of colonic IL-22 is induced under inflammatory conditions such as infection and IBD. Indeed, many of the IL-22-regulated proteins belong to the IBD susceptibility genes [50]. Mucosal IL-22 has a dual role in mediating mucosal healing (through activation of activation of STAT3 and pro-proliferative genes) and combating pathogens (via expression of AMPs). IL-22 is an essential cytokine in the fight against *C*. *rodentium* infection [12]. IL-22 is produced by ILC3 prior to 8 DPI and by Th-17/22 T-cells after 8 DPI; importantly, the abundance of various IL-22 producing cells (e.g. ILCs, Th22) was similar between uninfected, WT- and Δ*espO*-infected mice 8 DPI. The abundance of neutrophils recruited to the site of infection was the only difference we observed between WT and Δ*espO* at 8 DPI. The putative role of neutrophils as a source of IL-22 is supported by a recent report showing that depletion of neutrophils with anti-Gr-1 neutralizing antibody during *C*. *rodentium* infection resulted in significant reduction in IL-22 productions. The reduced number of neutrophils following infection with Δ*espO* was consistent with lower abundance of Mmp9 and was mirrored by a global decrease in expression of genes encoding AMPs, with the exception of Dmbt1 and Iod1. However, while calprotectin, Reg3β and γ, Lcn2 and Lyz2 are regulated by IL-22, Dmbt1 is mainly induced by IL-27 [51] and expression of Ido1is triggered by interferon gamma [52], suggesting that EspO selectively modulates innate immune responses in IECs. It is important to note that even though the abundance of the IL-22 regulated antimicrobial proteins is reduced following infection with Δ*espO*, the residual levels are sufficient to mediate bacterial clearance (albeit delayed), unlike *il-22* KO mice which succumbed to *C*. *rodentium* infection [12]. As Δ*espO* is shed for a longer period of time and triggers a novel immune response compare to WT (e.g. reduced abundance of T cells, neutrophils, IL-22 and *C*. *rodentium*-specific IgG) [23], the selective pressure for keeping EspO is not apparent. Our data show that by triggering damage repair responses EspO impacts on expression of antimicrobial peptides and the availability of trace minerals (e.g. Fe, Mg, Zn), which are required for survival by all living organism. While *C*. *rodentium* can resist nutritional immune responses and toxicity of antimicrobial peptides, these host responses affect the composition of the gut microbiota. Indeed, infection with Δ*espO*, which attenuates (yet not abolish) nutritional immunity, is associated with specific dysbiosis, particularly affecting the abundance of *Firmicutes*. Similarly, infection of mice lacking Mmp9, which is found in reduced abundance in Δ*espO*, with WT *C*. *rodentium* also resulted in increased abundance of *Firmicutes*, as well as *Lactobacilli* [53].

Although counterintuitive, the concept of bacterial T3SS effectors triggering inflammation is not new; indeed, multiple pathogens use this strategy as a means to disrupt both the epithelium and the microbiota in order to promote colonization [54]. T3SS effectors form a complex, yet robust, network that can resist sever perturbation (e.g. deletions). Alongside essential effectors (e.g. Tir, EspZ), *C*. *rodentium* encodes multiple accessory effectors, each making a refining contribution to the infection process. EspO is one such effector; its importance is emphasized by the fact that EHEC O157 contains two copies of the gene [22]. While we still do not know how EspO affects signaling (reflected by changing the abundance of 206 host proteins) in IECs, our data highlights a novel infection strategy involving activation of tissue healing responses as a means to trigger an advantageous nutritional immunity.

## Material and methods

### Bacterial strains and complementation

Wild type *C*. *rodentium* ICC169 (56) and ICC169 Δ*espO* (ICC1333) were grown at 37ºC in Luria–Bertani (LB) with necessary antibiotics. *espO* was amplified from ICC169 genomic DNA using primers GCTGGATCCTAGAAGAAGGAGATATACCATGCCATTGTCAATAAGAAA and GCTGTCGACTCAGGATTTATTTGAGTTATTAATCTCGGTC and was cloned in pACYC184 plasmid to generate pICC1379. The recombinant plasmid was confirmed by PCR and DNA sequencing (GATC Biotech).

### Ethics statement

All animal experiments complied with the Animals Scientific Procedures Act 1986 and UK Home Office guidelines and were approved by the Animal Welfare and Ethical Review Body (AWERB) at Imperial College London. The mouse experiments were performed under project licence PPL 70–8413.

### Oral gavage of mice and CFU count

Mouse experiments were designed in agreement with the ARRIVE guidelines [55] for the reporting and execution of animal experiments, including sample randomization and blinding.

Pathogen-free female C57BL/6 or Rag2^-/-^ il2rg^-/-^ mice (18 to 20 g) were inoculated by oral gavage with 200 μl of *C*. *rodentium* suspension (~5 x10^9^ colony forming units (cfu)). Uninfected mice were mock treated with PBS (200 μl). The number of viable bacteria used was determined by retrospective plating. Number of viable bacteria per gram of stool was similarly determined by plating onto LB agar.

### Tissue immunostaining and CCH measurement

Terminal colon (0.5 cm) was fixed in 10% neutral buffered formalin and paraffin-embedded. Paraffin-embedded sections were then treated as previously described^22^. Anti-intimin (a gift from Professor Fairbrother, Montreal University), E-cadherin (BD Biosciences) and Ki67 (Thermo Scientific) were used as primary antibodies followed by secondary antibodies from Jackson ImmunoResearch. H&E stained tissues were evaluated blindly for CCH by measuring the length of well-oriented crypts from each section from all of the mice. Similarly, Ki-67 staining was assessed microscopically by measuring the distance from the bottom of the crypt to the last stained nuclei. Ki-67staining was expressed as a ratio over the total length of the crypt. Tissues were imaged with an Axio, images were acquired using an Axio camera, and computer-processed using AxioVision (Carl Zeiss MicroImaging GmbH, Germany).

### Immunofluorescence staining of frozen sections

For neutrophils staining, indirect immunofluorescence was performed on cryo-sections as previously described [56]. Chicken anti-intimin and rat anti-Ly-6G (RB6-8C5; Santa Cruz) were used as primary antibody follow by secondary antibodies from Jackson ImmunoResearch. Images were acquired using an AxioCam MRm camera and processed using AxioVision (Carl Zeiss MicroImaging GmbH, Germany).

### Transmission electron microscopy

Murine colonic tissues were fixed in 2.5% (vol/vol) glutaraldehyde/PBS and processed for electron microscopy. Samples for transmission electron microscopy were observed using a Phillips 201 transmission electron microscope at an accelerating voltage of 60 kV (Philips, United Kingdom).

### Extraction of enterocytes

IEC have been extracted as previously described [18]. Cell pellets were either kept frozen for proteomic analysis, Western blotting or RNA extraction.

### Cell culture and infection

HeLa cells (ATCC) were maintained in low glucose Dulbecco's Modified Eagle Medium (DMEM) supplemented with Heat-inactivated fetal calf serum (10% vol/vol) (FCS, Gibco), 2mM GlutaMAX (Invitrogen), and 0.1 mM nonessential amino acids at 37°C under 5% CO_2_ atmosphere. *C*. *rodentium* was cultured in Luria Broth at 37°C, 200 rpm with appropriate antibiotics for 8 h and then subculture (1/500) in DMEM with low glucose and grown overnight at 37°C without agitation in 5% CO2 incubator. After 3 h of starvation in DMEM only, cells were infected for 3 h. HeLa cells were incubated with IL-6 (Biovision, 50ng/ml) for 30 min. prior to analyzing cell extracts by Western blotting, using Hax-1 as a loading control.

### Western blotting

IECs or HeLa cells were lysed in 50 mM Tris pH 7.4, 150 mM NaCl, 2 mM EDTA, 1% NP-40 and 1% SDS. Following gel electrophoresis and transfer, membranes were washed with PBS 0.1% Tween, blocked in TBS (0.1% Tween, 3% BSA, 0.5% gelatin) and probed with specific antibodies overnight. Blots were then incubated with secondary antibody (Jackson ImmunoResearch), followed by EZ-ECL assay, according to the manufacturer's instructions (Geneflow). Chemiluminescences were detecting using a Chemidoc (Biorad). Polyclonal anti-GADPH (Abcam), anti-Hax1 (Genetex), anti-Stat3 and monoclonal anti- phopho-Stat3 (Cell Signaling) were used to detect the different proteins.

### Isolation of mRNAs and qRT-PCR

Enterocytes mRNAs were isolated using a RNeasy minikit according to the manufacturer's instructions (Qiagen). Samples were treated with RQ1 DNase I and reverse transcription was perform using RQ1 DNase I according to the manufacturer's instructions (Promega). Targeted genes were amplified with specific primer pairs listed in S3 Table, using a 7300 Applied Biosystems instrument under standard cycle conditions for PowerUp SYBR Green Master Mix (Thermo Fisher). Changes in gene expression levels were analyzed relative to the controls (uninfected samples), with GAPDH as a standard, using the ΔΔ*C*_*T*_ method.

### Explant and IL-22 ELISA

Last centimeter of distal part of the colon has been excised, weighted and washed thoroughly with RPMI medium with 100ug/ml of streptomycin and 100U/ml penicillin. Tissues have been then culture in RPMI (10%FCS, P/S, L-Glu) for 2h and placed in fresh media (0.1 ml / 10 mg of tissue). After 24h, the supernatant has been collected and centrifuged 15 min at 15000 rpm. IL-22 was then measured using Mouse IL-22 DuoSet ELISA (R&D Systems) according to manufacturer's instructions.

### Lcn2 ELISA

Fresh stool pellets were resuspended in PBS-0.1% Tween20 at a w/v ratio of 100 mg of stool per 1 ml PBS. Samples were left shaking for 20 min, centrifuged at maximum speed for 10 min before freezing the supernatant. Fecal LCN-2 was then measured using Mouse Lipocalin-2/NGAL DuoSet ELISA (R&D Systems) according to manufacturer's instructions.

### Mass spectrometry

IEC pellets isolated from WT, Δ*espO* and ΔespO-pespO *C*. *rodentium* infected and uninfected mice were analyzed as previously described [18]. Only unique peptides were used for quantification, considering protein groups for peptide uniqueness. Peptides with average reported S/N>3 were used for protein quantification. The IEC obtained from uninfected mice were used as controls for log2 ratio calculations. Differential expression p-values were computed based on a single-sample t-test. Specificity thresholds used to characterize the EspO-specific IECs proteins were define as p-value < 0.05 (Student's *t*-distribution) and log2 ratio > 0.59 or < -0.59 (equivalent to 1.5 fold change) compare to protein abundance following WT and ΔespO-pespO infections. Specifically regulated protein were uploaded in Ingenuity Pathway Analysis (IPA) (Qiagen) platform. Trends of activation/inhibition states of the enriched functions and regulators were inferred by the calculation of a z-score (-2 < z-score > 2).

### 16S rRNA gene sequencing

Colons were collected from mice and DNA was isolated using PowerSoil DNA Isolation Kit (MO BIO Laboratories). For 16S amplicon pyrosequencing, PCR amplification was performed spanning the V3and V4 region using the primers 515F/806R of the 16S rRNA gene and subsequently sequenced using 500bp paired-end sequencing (Illumina MiSeq). Reads were then processed using the QIIME (quantitative insights into microbial ecology) analysis pipeline with USEARCH against the Greengenes database.

### Isolation of cells from intestinal tissue and ex vivo stimulation

Cells were isolated from LI LP as previously described (53) using a digestion solution containing 25 μg/mL liberase TL (Roche) and 25 μg/mL Dnase1 (Sigma Aldrich). For cytokine ex-vivo stimulation, 1–5 x 10^6^ cells were incubated at 37°C with IL-1β (R&D Systems; 100 ng/mL), IL-23 (R&D Systems; 100 ng/mL), PMA (Sigma-Aldrich; 50 ng/ml), Ionomycin (Sigma-Aldrich; 2.5μg/ml) and BD GolgiPlug (BD Biosciences) in 10% FCS DMEM (Gibco) for 3h.

### Flow cytometry

Single-cell suspensions were stained with Flexible Viability Dye eFluor 506 (eBioscience) and blocked with FcR Blocking Reagent (Miltenyi) for 15 minutes followed by 30 minutes of surface antigens staining with a combination of fluorescently conjugated monoclonal antibodies (from BD Biosciences, eBioscience and Biolegend) on ice. For experiments involving intranuclear transcription factor staining, cells were fixed, permeabilized and stained using Fix & Perm Buffer Kit according to the manufacturer’s instructions (BD Biosciences). For intracellular cytokine staining, cells were fixed, permeabilized and stained using Fix/Perm kit according to the manufacturer’s instructions (BD Biosciences). All the samples were acquired on a custom-configuration LSR Fortessa (BD Biosciences) and the data were analyzed on FlowJo10 software (TreeStar).

### Statistical analysis

GraphPad Prism software was used for all statistical calculations. Statistical test used was Mann-Whitney compared to controls (or as indicated in the figure). p-values < 0.05 were considered significant. For the microbiota, p-values were FDR corrected using Benjamini and Hochberg method.

## Supporting information

S1 Fig The A/E lesion signature of *C*. *rodentium* infection. **(A)** Bar plot showing the relative abundances of the individual proteins within the BB network in the IEC infected with WT.
**(B)** Bar plot showing the relative abundances of the individual proteins within the BB network in IEC infected with Δ*espO* compared to WT.
(TIF)

S2 Fig Flow cytometry analysis. Flow cytometry analysis of colonic lamina propria lymphocytes after *C*. *rodentium* infection 8 DPI.
(TIF)

S1 Table Abundances (Scaled) of *C*. *rodentium* proteins. (XLSX)

S2 Table Log 2 fold change of the EspO specific proteins. (XLSX)

S3 Table List of primers for qPCR. (DOCX)
