# Supplementary figures and images for: The Citrobacter rodentium type III secretion system effector EspO affects mucosal damage repair and antimicrobial responses
Source: PLoS Pathog. 2018 Oct 26;14(10):e1007406. doi: 10.1371/journal.ppat.1007406 (PMC6221368; doi:10.1371/journal.ppat.1007406)

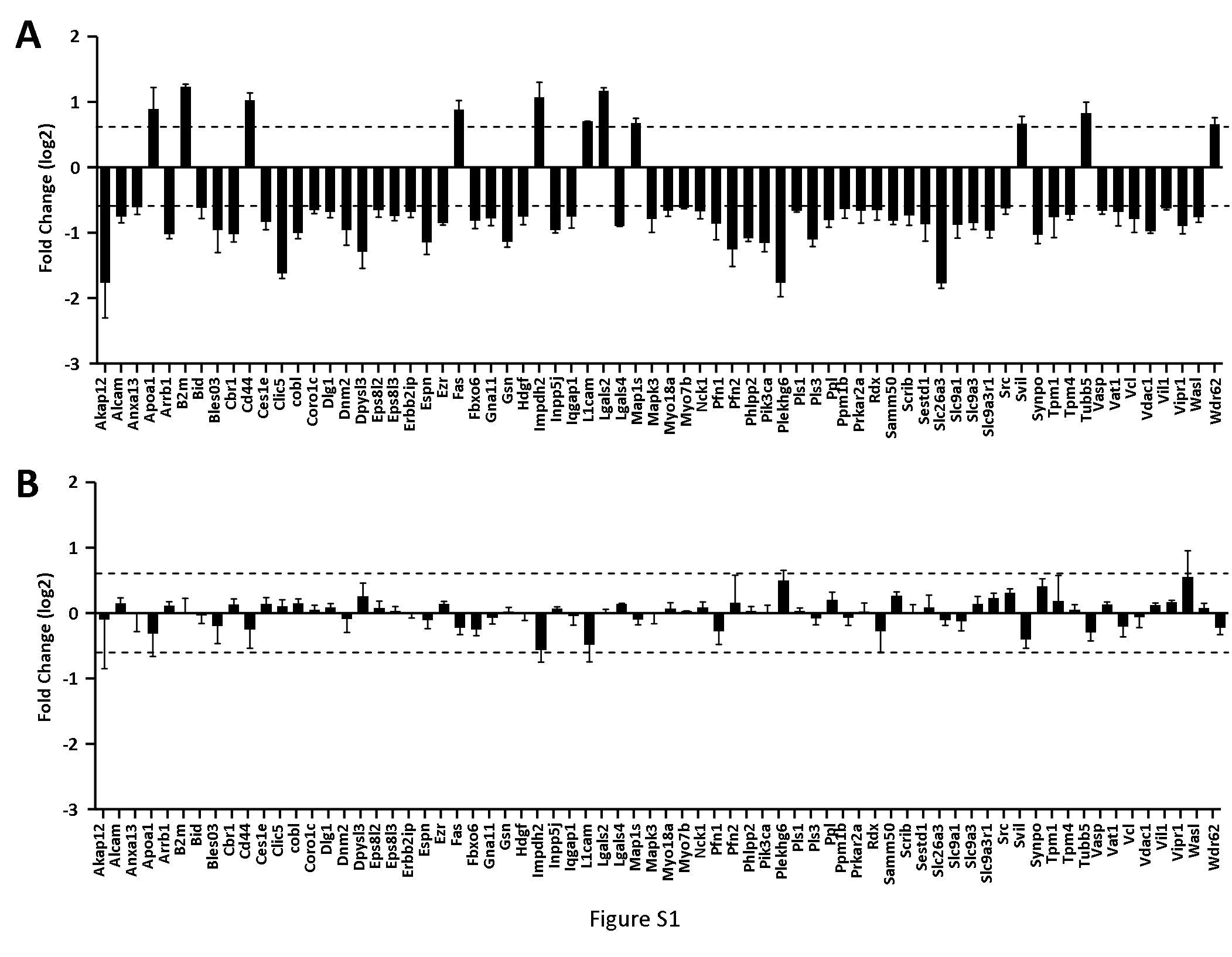

Supplement: S1 Fig — (A) Bar plot showing the relative abundances of the individual proteins within the BB network in the IEC infected with WT. (B) Bar plot showing the relative abundances of the individual proteins within the BB network in IEC infected with ΔespO compared to WT. (TIF) [file ppat.1007406.s001.tif]

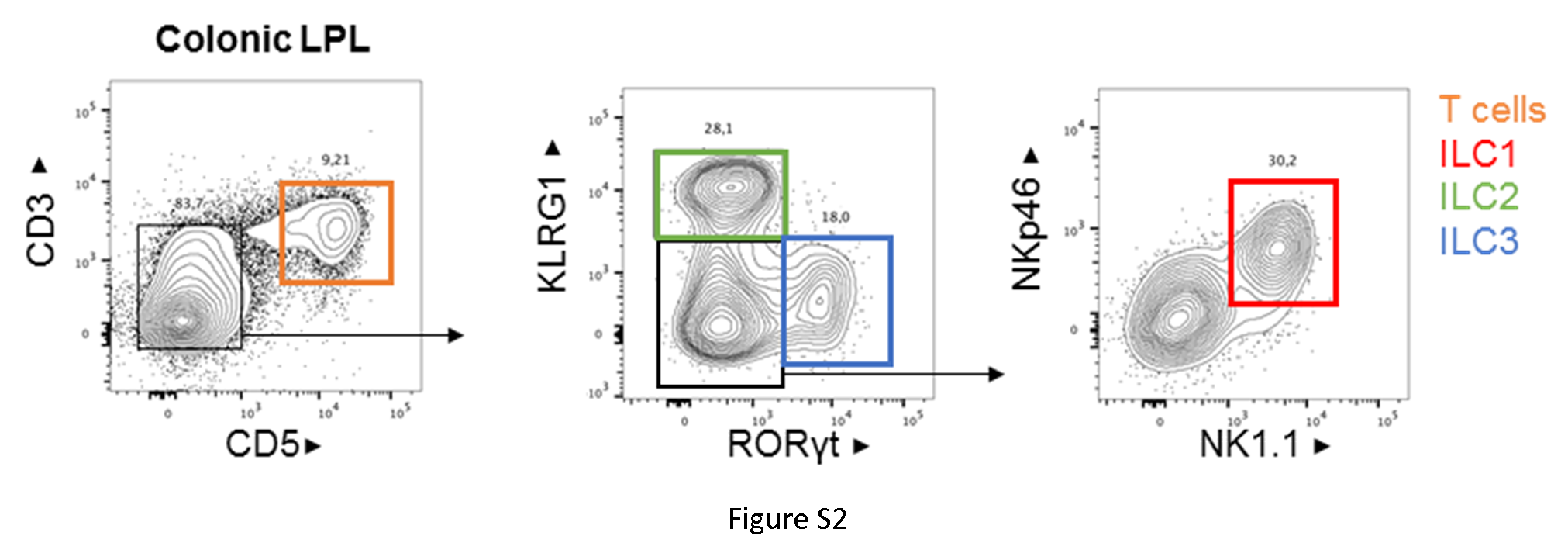

Supplement: S2 Fig — Flow cytometry analysis of colonic lamina propria lymphocytes after C. rodentium infection 8 DPI. (TIF) [file ppat.1007406.s002.tif]
