# Supplementary material for: The Citrobacter rodentium type III secretion system effector EspO affects mucosal damage repair and antimicrobial responses
Source: PLoS Pathog. 2018 Oct 26;14(10):e1007406. doi: 10.1371/journal.ppat.1007406 (PMC6221368; doi:10.1371/journal.ppat.1007406)
Supplement: S3 Table — (DOCX) [file ppat.1007406.s005.docx]

**Supplementary figure legends and tables**

**Figure S1. The A/E lesion signature of *C. rodentium* infection.**

1. Bar plot showing the relative abundances of the individual proteins within the BB network in the IEC infected with WT.
2. Bar plot showing the relative abundances of the individual proteins within the BB network in IEC infected with Δ*espO* compared to WT

**Figure S2. Flow cytometry analysis**

Flow cytometry analysis of colonic lamina propria lymphocytes after *C. rodentium* infection 8 DPI.

**Table S3. List of primers for qPCR**

| Target | Direction | Sequence 5’-3’ |
| --- | --- | --- |
| Reg3β | Forward | TGGTGAAGAGAACAGGAAACAG |
|  | Reverse | GGCAGTAGATGGGTTCCTC |
| Reg3γ | Forward | TTACATCAACTGGGAGACGAATC |
|  | Reverse | GGCCTTGAATTTGCAGACATAG |
| Dmbt1 | Forward | TGGAGGCTATGAGGACTATCTG |
|  | Reverse | TGGTTTGGTCAGTTGGGTAG |
| Ido1 | Forward | CAATCAAAGCAATCCCCACTG |
|  | Reverse | AAAACGTGTCTGGGTCCAC |
| Cxcl-1 | Forward | AACCGAAGTCATAGCCACAC |
|  | Reverse | CAGACGGTGCCATCAGAG |
| Ifn-γ | Forward | ATGCATTCATGAGTATTGCCAAG |
|  | Reverse | ACTCCTTTTCCGCTTCCTG |
| IL-22 | Forward | AGCTTGAGGTGTCCAACTTC |
|  | Reverse | GGTAGCACTGATCTTTAGCACTG |
| Gapdh | Forward | TCAACAGCAACTCCCACTCTTCCA |
|  | Reverse | ACCCTGTTGCTGTAGCCGTATTCA |
